# Conceptualizing work-related mental distress in the British coalfields (c.1900–1950)

**DOI:** 10.1057/s41599-018-0187-4

**Published:** 2018-11-06

**Authors:** Vicky Long, Victoria Brown

**Affiliations:** 1 Newcastle University, Newcastle, UK; 2 Independent researcher, Glasgow, UK

## Abstract

The history of occupational health is now an established and growing field of study, yet to date comparatively little research has been undertaken on the history of work-related mental health issues, which currently account for 40% of all work-related illnesses in the UK. Work-related stress is usually seen as a post-war and post-industrial phenomenon, largely confined to white collar and service sectors, which developed as a consequence of changing working cultures. However, through a close reading of diverse source sets generated by, or pertaining to, the coal industry, this article unearths another history of work-related mental health issues; a history focussing on a heavy industry in the early years of the twentieth century. Through this analysis, we argue that the heavy industries more typically associated with physical illnesses and disabilities also generated mental distress amongst their workers, which reflected the distinctive pressures of these working environments. The article explores how understandings of the mental distress experienced by miners were shaped by medical, political, economic, occupational and industrial factors, and generated debates between different organizations as to the cause of these disorders and appropriate responses. Further analysis of work-related mental disorders in different occupational sectors may allow researchers to tease out the nuanced role played by workplace environments and working practices on mental health, and to examine the relationship between physical and mental health at work.

## Introduction

In 2010, the plight of 33 miners trapped underground in Chile after the collapse of a tunnel attracted global media attention. Given the dramatic contraction of coal mining in Britain, the story served as a powerful reminder that the physical hazards of heavy industry have been relocated rather than eradicated, as Christopher Sellers and Joseph Melling have argued (2012a, 2012b).Yet this story also asks us to rethink conventional narratives about work-related mental illnesses.

The changing profile of occupational morbidity in the UK has typically been located within the transformation of the labour market; with the demise of the industrial sector in the UK, we have witnessed a corresponding decline in physical illnesses and disabilities linked to work. Meanwhile, commentators have linked the surge in mental health issues to the rise of the service sector and changing workplace cultures, such as longer working hours, zero hour contracts, and a blurring of boundaries between home and work (Grimshaw, 1999; Bunting, 2004; Wainwright and Calnan, 2002). Within Higher Education, for example, rising stress levels amongst lecturers have been attributed to a mounting workload, loss of control over the working environment and job role, and poor management (Devonport et al., 2008). This narrative effectively accounts for the prevalence of work-related mental distress in contemporary working environments in Western Europe and America, but we believe that it conceals other histories of work-related mental distress. While the story of the Chilean miners initially appeared to have a happy ending when all 33 miners were rescued alive 69 days later, nine of the miners were subsequently reported to be suffering from post-traumatic stress (Barrionuevo, 2011).

Mining has always been, and remains, a physically hazardous industry, although the precise nature of these risks has been shaped, amongst other things, by geology, workplace technologies, working practices and cultures, and the extent of health and safety measures in place at any given point and place in time. While the number of workers killed in industrial accidents in the UK fell by more than a half between 1914 and 1950 (McIvor, 2013, p. 132), health and safety within the coal industry stagnated; hit heavily by the interwar depression, companies failed to maintain mines and equipment, while electrification and mechanization introduced new accident risks and raised dust levels within mines, heightening the risk of pneumoconiosis. Moreover, miners’ power, and consequently their ability to control their working environment, declined as the industry contracted (McIvor and Johnston, 2007, pp. 43-45). It is not surprising that histories of occupational health in mining have focussed upon the physical harm which mining wrought upon the bodies of workers: the deaths, illnesses, injuries and physical disabilities caused by accidents, working practices, and exposure to toxic dust (McIvor and Johnston, 2007; Mills, 2010; Bufton and Melling, 2005; Wallace, 1987; Curtis and Thompson, 2015; Turner and Blackie, 2018). Indeed, the historiography of occupational illness in general has focused on the risks posed by work to physical as opposed to mental health.

Drawing on a diverse range of source materials which were generated by or pertain to coal mining and coal miners, most of which were gathered while researching physical disability in the coalfields, the aims of this article are twofold: it seeks to illustrate the kinds of source and methods which could help construct a broader, more inclusive history of work-related mental distress, and it demonstrates how we have applied this approach to commence studying the history of mental distress in coal mining. Our goal is not to contribute to understandings of the rise of occupational stress, but to chart earlier manifestations and understandings of work-related mental distress, which were shaped by social, political and industrial circumstances, and consequently differs materially and conceptually from what we term occupational stress today. Therefore our analysis focuses on the first half of the twentieth century, an era characterized by contraction of the coal industry and erosion of health and safety provisions in the interwar years. This period was bookended by the 1897 Workmen’s Compensation Act, which enabled employees incapacitated through workplace accidents and, later, occupational illnesses, to secure compensation from their employers (Bartrip and Burnam, 1983; Bartrip, 1987), and which, we argue, played a significant role in shaping, and indeed, repressing, experiences of work-related mental distress; and the identification of occupational stress in the post-war era, a phenomena which has attracted significant scholarly work. Where appropriate, we integrate materials pertaining to work-related distress which pre- or postdates this period, but sheds light on the pattern of work-related distress in the heavy industries examined here, as opposed to what became termed occupational stress.

We start by summarizing the existing historiography on work-related mental distress. The article then considers the methodological challenges of researching the history of work-related mental distress amongst miners, and the broader challenges of studying work-related distress amongst different occupational groups. The main body of the article provides a detailed examination of some of the cases of mentally-distressed miners we encountered in our research. We will use these examples to argue that the physical hazards of coal mining—most notably, its high accident rate, but also the illnesses associated with mining—appear to have played a significant role in triggering mental distress amongst miners. In this sense, physical and psychological occupational disorders are not distinct entities, but are intimately connected. Arthur McIvor, who has used oral history testimony to gauge the impact of physical illness and disability on workers, recently observed that ‘the effects upon mental health could be as devastating—sometimes more so—than the physical impairment…This emotional history has hardly been explored’ (2013, pp. 192–193). Our research indicates that unemployment, and fear of becoming unemployed, also played a role in causing mental distress. These two factors—accidents and unemployment—sometimes intersected in cases where miners feared that their future employment prospects had been compromised by physical incapacity arising from a workplace accident or occupational illness.

Finally, we will argue that the low visibility of work-related mental disturbance in mining does not automatically equate to a low incidence. Rather, the prioritization of tangible risks to physical health marginalized understandings of, and responses to, work-related mental distress. Indeed, the difficulty of identifying distressed miners in archival records reflects the fluid and contested nature of work-related distress. Although we use the term distress in this article, the lexicon which in practice identifies the parameters of our field of study is varied, encompassing neurosis, fear, trauma, stress, post-traumatic stress disorder, malingering and absenteeism. This terminology reveals how conceptualizations of work-related mental distress have been constructed, embodying social values and cultural beliefs about class and gender, as well as reflecting changing medical ideas. It also reflects the fact that we are dealing with a range of emotional expressions and experiences. Accessing this experiential history is, however, elusive: our sources frequently demonstrate the scale of the issue, but rarely allow us to interrogate it in depth.

## Histories of work-related mental distress

The historiography on work-related mental distress focuses on two areas: the development of industrial psychology within manufacturing in the early twentieth century, where unskilled repetitive work was seen as a threat to mental wellbeing, and the history of stress amongst white collar workers in the post-war era. Government intervention during the course of the First World War—namely the establishment of the Health of Munition Workers Committee, intended to maintain industrial productivity by preserving workers’ health, and its successor organizations, the Industrial Fatigue and Industrial Health Research Boards—indirectly propelled a growing interest in psychological factors, as the emerging specialisms of industrial welfare and industrial psychology gradually displaced the wartime focus on physiology and industrial fatigue (Long, 2011). By the interwar era, individuals from across the political spectrum expressed the view that monotonous and repetitive factory work had a detrimental psychological impact upon workers. As the size of workplaces and workforces increased, the production process was subdivided and mechanized. Workers undertook increasingly specialized, unskilled, repetitive tasks; boredom and alienation resulted. Even the Assistant Secretary of the Trades Union Congress (hereafter TUC), a body established in 1868 to coordinate trade union interests in England and Wales, argued that ‘a good deal of industrial unrest is due as much to mental revolt against the monotony and lack of variety in modern industrial production as it is to evils relating to wages or hours’ (Bramley, 1921, p. 408).

A number of researchers interested in the development of psychological expertise and the permeation of psychological knowledge into disparate social spheres have shown how industrial psychologists in the interwar years sought to foster productivity within factories by maximizing the fit between individual workers and their work (Hayward, 2009; Miller, 1986; Rose, 1989; Thomson, 2006; Whitelaw, 2009). Work, argued practitioners of industrial welfare and industrial psychology, should be tailored to the innate skills and temperament of individual employees. In practice the preponderance of unskilled, repetitive work made this difficult, and individual workers tended to be categorized into personality types with perceived common traits, thereby homogenizing individuality (Long, 2011, pp. 130–155). Despite the body of research undertaken during the First World War and interwar period which demonstrated that long working hours were counterproductive, industrial production during the early years of the Second World War was characterized by deregulation of working hours and a surge in reported workplace accidents (Long, 2011, pp. 37–40). In his wartime study of factory work undertaken for the Industrial Health Research Board, Dr Russell Fraser claimed that 10 percent of workers were suffering from a definite and disabling neurosis, and a further 20% experienced minor neuroses. In his view, neurotic illness accounted for one quarter to one third of all illness absence from work, even though it was not described as such (1947).

In the post-war era, some researchers viewed the increasing automation of industrial processes as a threat to workers’ psychological wellbeing, although other commentators considered these developments as a means of liberating workers from mundane tasks (Hayes, 2015). Industrial psychology also shaped new theories on the origins and consequences of industrial accidents in the early twentieth century. John Burnham, for example, has discussed how psychologists identified the ‘accident prone’ individual, a concept which apportioned blame to workers, rather than risky environments (2009). Meanwhile, the notion that accidents and events could precipitate psychological trauma coalesced in the early decades of the twentieth century, fuelled in no small measure by doctors’ conceptualization of shellshock (Micale and Lerner, 2001).

The concept of occupational stress evolved from the scientific elaboration of stress in the early- and mid-twentieth-century as a maladapted physiological response to external stressors, a process which Mark Jackson reveals was forged as much by political and cultural factors as it was by medical and scientific knowledge (2013). Jackson demonstrates how assumptions about class, gender and personality informed the permutation of occupational stress which began to be articulated in the 1950s and 1960s, as concerns about post-war reconstruction, automation and failing businesses focused attention on the relationship between work, health and productivity. While researchers acknowledged that workplace factors played a role in the production of stress, many argued that personality traits were the primary causal factor, and claimed that rates of stress were higher amongst women and lower-grade workers. Despite this, research tended to focus on stress in white-collar occupations, a trend which Jackson suggests reflected the tendency to prioritize the health and wellbeing of the leaders of industry (ibid: 198–210). Debbie Palmer’s study of late twentieth-century surveys of civil servants’ health supports Jackson’s contention that perceptions of class, gender and personality shaped interpretations, revealing how a 1967 survey accounted for disproportionate rates of sick leave in some areas by blaming workers’ personal failings and the genetic inferiority of lower-grade workers. While a later study led by epidemiologist Michael Marmot emphasized the influence of external social structures and how these generated health inequalities, Palmer observes that this has done little to dispel the belief that occupational stress and its management is primarily a matter of ‘individual weakness and personality responsibility’ (2015, p. 109). Surveying contemporary publications, James Davies similarly concludes that mental health charities are complicit in depoliticizing work-related mental distress by focussing on the affected individual and ignoring the wider social and political factors at work (2016).

In recent contributions to the literature, Joseph Melling argues that the main driving force behind the growing acceptance of occupational stress as a phenomenon was not the evolution of scientific thought, but the transformation of the global economy which reworked industrial relations, leading to the erosion of collective bargaining and a growing individualization of work (2014; 2015). This might appear to support the dominant narrative of the rise of occupational stress, but Melling’s objective is not to chart the emergence and rise of occupational stress as an embodied entity, but to examine the ‘social, institutional, and intellectual constraints’ which inhibited discussion of occupational stress before 1970 (2014, p. 214). One crucial factor in this respect, he concludes, was the introduction of workmen’s compensation legislation in the late nineteenth century. Initially designed to compensate those incapacitated via workplace accidents, this legislation was expanded to cover workers suffering from certain occupational illnesses (Bartrip and Burman, 1983; Bartrip, 1987); similar provisions were enacted across Europe throughout the late nineteenth century (Moses, 2018). Drawn in to provide medical evidence in adversarial compensation battles between employers and employees, many doctors attributed rising compensation claims to malingering, or else argued that neurotic and unstable individuals were unconsciously simulating physical symptoms. It was not the manual labour undertaken by the working-classes, Melling argues, but the intellectual work of the middle-classes which doctors viewed as a potential threat to mental wellbeing. As we shall see, these understandings did much to render experiences of mental distress amongst coal miners invisible.

## The challenges of locating the mentally distressed miner

Surveying the types of records where one might expect to find mentally distressed miners is initially unpromising. Physical trauma is everywhere, but psychological distress seems absent. In all likelihood, however, this reflects a tendency to conceal mental distress—a tendency which in part stemmed from the working culture generated by miners themselves. Jamie Bronstein observes how newspaper reports of industrial accidents in the nineteenth century constituted a ‘pornography of death’, in which bodily mutilation was laid before readers in vivid, gory detail (2007, p. 72). Workers’ emotional responses are strikingly absent from such accounts, yet this does not prove the absence of emotion. Indeed, Bronstein suggests that such reports were framed in the language of worker heroism, and that workers’ ability to graphically recall such accidents many years later in fact reveals the considerable emotional impact of these events, although workplace cultures encouraged workers to repress these emotional responses. David Turner and Daniel Blackie have identified some eyewitness accounts of nineteenth-century mining disasters which describe survivors who display signs of trauma and psychological shock, but observe that these detail only the treatment of survivors’ physical injuries (Turner and Blackie, 2018, pp. 71–72). Heavy and hazardous industries, argue Ronnie Johnston and Arthur McIvor, functioned as sites for the construction of a ‘hard man’ machismo culture, in which workers—partly to demonstrate their masculinity, and partly to maximize their wages and protect their jobs—took risks at work (2004). Older miners helped perpetuate this culture, encouraging younger miners to avoid displays of emotion (Campbell, 2000, p. 238). As Ali Haggett has recently argued for the post-war era, this construction of masculinity has militated against openly discussing mental illness at work (2015, pp. 67–81).

What about sources which might allow us to quantify rates of mental distress amongst miners? Friendly society records, which historians have found to be a fruitful source for examining patterns of occupational morbidity, contain relatively few cases of mental disturbance. Yet, as James Riley argues, this may well reflect the decision taken by many friendly societies to refuse to pay benefits for insanity, and the likelihood that many psychological disturbances were consequently attributed to physical illnesses (1997, pp. 140–142). Indeed, correspondence from friendly societies to the Home Office in 1870 suggests that some were uncertain as to whether insanity was an illness or not, and therefore whether it was even legal to pay sickness allowance in cases of insanity. ‘Several societies have stopped the pay of their members as are so afflicted to prevent legal proceedings being taken against them’, explained the secretary of the joint committees of the friendly societies of Hastings and St Leonards. ‘It is needless to say that such stoppage is working great hardship on the wives and families of those members when they are least able to bear them’ (Richardson, 1870). A similar picture emerges if we turn to consider sickness certification. Anne Digby found a very low proportion of cases of mental distress in a set of interwar National Health Insurance records from Glasgow (1999, p. 209). This does not, however, preclude the possibility that other diagnostic terms were being applied to such patients. Indeed, when interviewing GPs who worked in the post-war era, Ali Haggett found that many doctors colluded with patients to exclude diagnoses suggestive of mental disorder when certifying patients as unfit for work, hoping to spare their patients from stigma. ‘Because the man didn’t want to be labelled as psychological, the doctor would go along with it’, observed one professor of general practice (2015, p. 69).

Haggett concludes that psychological illness amongst working men in the post-war era tended to manifest as psychosomatic illness—notably, gastric trouble—evading detection by occupational health researchers (2015, pp. 57–81). Evidence from the operation of provisions established under the auspices of the Disabled Persons (Employment) Act of 1944 points to a similar conclusion. This legislation created industrial rehabilitation units to restore disabled people’s working capacity; obliged larger employers to hire registered disabled staff; introduced disablement resettlement officers to place people into employment, and provided sheltered work opportunities. Examining 68 consecutive entrants to Hillington industrial rehabilitation unit in 1949, psychiatrists Allan C. Tait and T. Ferguson Roger found that while five had been referred as cases of psychological illness, a further ten were primarily cases of psychiatric illness (1950). Other publications appeared to corroborate these findings. Mark Hewitt, a medical adviser to the Ministry of Labour and National Service, analysed 50 men registered as disabled in Manchester. While only four of these men had been diagnosed with a psychological disability, Hewitt believed that 17 had a psychological disability, and a further 26 were disabled through a combination of physical and mental factors. ‘There was a tendency for the disability label to be altered and for mental disability to assume an organic title’, he concluded (1949, p. 523). An article examining the Leicester industrial rehabilitation unit published in the same year observed that entrants fell into two categories: those ‘who have passed the convalescent stage of some disorder but are left with a residual disability, often only psychological’, and those with ‘some long-defect’ who had ‘gradually become unable to work satisfactorily in industry.’ The author concluded that more than half of the individuals in both categories suffered from some form of neurosis or psychopathy (Anonymous, 1949, p. 999).

Workers’ convalescent homes predated the state-run industrial rehabilitation units, and also sought to restore people’s working capacity after accident and illness. They often gave the impression that they did not cater for mentally distressed workers. Conishead Priory Convalescent Home, for example, which provided for miners in Durham County, refused to admit ‘helpless persons of unsound mind’ (Durham District Welfare Committee, 1929). However, this definition seems to refer only to individuals suffering from serious mental illness, potentially leaving the door open to people suffering from milder, or less noticeable forms of mental distress. In her work on Scottish convalescent homes, Jenny Cronin suggests that such patients may have been admitted as suffering from nervousness or debility (2003, p. 165).

Terminology, in other words, was consistently and knowingly exploited to obscure work-related mental distress. Even researchers who elected to study work-related mental distress engaged in this subterfuge. The Occupational Psychiatry Research Unit, set up by the Medical Research Council in 1948 at the request of the psychiatrist Professor Aubrey Lewis, was renamed the Unit for Research in Occupational Adaptation in 1951, because some members of the unit found the word ‘psychiatry’ embarrassing when contacting companies. ‘The essential thing is obviously to have a title which does not contain the words “psychiatry” or “mental”, because of their associations’, explained Lewis (1951). This trend of concealment makes it extremely difficult to chart the extent of work-related mental illness with any certainty, a task further complicated by the difficulty of disentangling the impact of work factors and external factors in producing mental distress. Indeed, while mental illness might often be concealed, there could be instances in which it would be financially advantageous for miners or their families to try and connect work and mental illness—to secure compensation, for example.

The above discussion demonstrates that it is possible to locate the mentally distressed miner in a diverse range of source materials. However, given the difficulty of identifying work-related mental distress in some of the types of records we would typically turn to when researching occupational health, this article will focus on records generated to document mental distress. By looking at a few examples, we can examine how agents responded to, and contested, work-related mental distress; we can explore whether the physical hazards of coalmining generated psychological consequences, and we can examine whether efforts were made to afford workers medical care, rehabilitation, and financial support by attributing mental distress to physiological causes.

So let us start with asylum records, where recent historical research has begun to explore the extent to which medical and lay commentators viewed work-related factors as a cause of mental breakdown. Psychiatrists were more inclined to view overwork as a cause of mental breakdown in middle-class as opposed to working-class patients (Melling, 2014; Melling, 2004; Suzuki, 2007), and Alice Mauger notes that work and finance was the leading moral cause listed for male patients in admissions documentation for fee-paying asylum admissions in nineteenth-century Ireland (2018, p. 153). Yet within Irish pauper asylums in the same era, medical officers also recognized that adverse economic circumstances could have a detrimental impact on patients’ mental states, viewing men’s failure to provide for their families in particular as a cause of insanity (Cox, 2012, p. 121). And while psychiatrists may have been less inclined to attribute the breakdowns of working-class men to work-related causes, lay interpretations could differ. Using admissions records for Hanwell asylum in the nineteenth century, for example, Akihito Suzuki demonstrates that many relatives attributed men’s breakdowns to overwork, anxiety about long-term employment prospects and fear of financial ruin (2007, pp. 118–128).

Indeed, while psychiatrists themselves were reluctant to attribute working-class men’s breakdowns to their work, admissions documents and case notes often paint a different picture. Analysing the records of slate quarrymen admitted to Denbigh asylum, Pamela Michael argues, reveals the physical and psychological impact of their work (1997). Locating coal miners in the case books of asylums servicing coalfield areas in Britain, we found examples in which physical injuries arising from mining accidents appeared to trigger mental disturbance. Take Joseph Barber, for example, who was admitted to Newcastle Asylum in 1896 suffering from mania. Joseph, a former miner aged 66, was described as ‘restless, wanting to go out to his work in the middle of the night.’ He had not worked since he hurt his back and broke his thigh and ribs in a pit accident three and a half years earlier (St Nicholas Hospital). Meanwhile in 1912, a 36 year old miner named Patrick Curran was admitted to Woodilee Asylum near Glasgow, described as suffering from confusional insanity (Woodilee Hospital). Patrick’s father had been killed in a coal mine aged 36, and Patrick himself had been involved in a pit accident at Prestonpans in 1909, when his spine was injured by a fall of coal. This, according to his mother, triggered his illness. Following treatment at Edinburgh infirmary, Patrick was admitted for a spell at Haddington Asylum. He had also started to consume alcohol heavily at weekends. His mother told the doctor that her son had ‘never been sane since the accident.’

Our argument is not that coalminers were disproportionately represented within asylums; after all, John Walton found that coal miners were underrepresented in admissions to Lancashire asylum in the mid nineteenth-century (1979). Rather, we suggest that qualitative analysis of asylum records may provide some insight into miners’ experiences and emotional states. Admittedly, this insight is mediated by the perspectives of the recording doctor or the family member providing information, and case notes are uneven in terms of availability and what they record. Yet, as Hilary Marland observed, ‘we would poorly serve the majority of nineteenth-century psychiatric patients… if their case histories were ignored, for we have little else to work with’ (2004, p. 101). Equally, these sources offer us insights into the interpretive models deployed by miners’ families and doctors to account for mental breakdown.

Accidents appear to have been a significant factor in both Joseph Barber and Patrick Curran’s journeys into asylums. Let us turn to another case of a miner injured in an accident, retrieved in paperwork generated by the National Insurance (Industrial Injuries) Act. This piece of legislation supplanted earlier workmen’s compensation legislation which dated back to the late nineteenth century, and provided compensation for industrial accidents, illnesses and deaths (Bartrip, 1987). Local insurance officers dealt with cases, and the claimant could appeal decisions at local appeal tribunals. If the claimant appealed the decision of the local tribunal, the Commissioner or Deputy Commissioner ruled on the case. The adversarial nature of these proceedings often prompted fierce debate as to the cause and nature of the worker’s incapacity or death (Turner and McIvor, 2017). In turn, these debates illuminate understandings of the boundaries between physical and mental illness, the ways in which social values shaped interpretations of miners’ mental distress, and the impact indeed of the mechanisms of compensation on miners’ states of mind.

On 31 March 1955, Walter A. had been working in a low seam when a deputy fired a shot into a low seam on a long wall face, in breach of regulations (Ministry of Pensions, 1955–1959). Walter had been in front of the deputy and around five yards from the wall when the shot was fired, and the force of the explosion knocked him over. He sustained multiple lacerations to his face and shoulders from flying coal particles which resulted in scarring, and received injury benefit until August 1955 when a medical board subsequently awarded him 3% disablement benefit for life on the grounds of disfigurement. Walter subsequently appealed the 3% award on the grounds that he was suffering from traumatic neurosis as a consequence of the accident, and had lost earnings. Although he had resumed work—first on the surface, and then at the bottom of the pit—his symptoms prevented him from resuming his former occupation. These included daily spells of dizziness; a heavy feeling in his right arm; seeing lights before his eyes, and extreme insomnia, alongside nervousness and depression. An appeals tribunal rejected Walter’s appeal for a 40% disablement benefit, arguing that there was no organic basis for the neurosis, although Sir Archibald Safford, the Commissioner presiding, subsequently ruled in favour of Walter. ‘It does not require much imagination to appreciate that to be within five yards of a shot being fired at the coalface while in a low seam must have been a terrifying experience’ he observed. The branch secretary of the National Union of Mineworkers informed a subsequent appeal that there had been 12 such accidents at the colliery in the past 15 years, and that not one of the men injured in these accidents had returned to work at the coal face.

How often did miners who experienced traumatic accidents simply not return to work? At a witness seminar held by the Scottish Oral History Centre at Strathclyde University, Nicky Wilson, the current leader of the National Union of Mineworkers in Scotland, recalled the explosion at Cardowan colliery in 1982 that sent a fireball up and down a tunnel. Forty men sustained superficial burns, but escaped major injury. The union secured compensation for these men, but none of them ever worked underground again. Some, Nicky recalled, became alcoholics, while one committed suicide. ‘If you witness someone being killed’, explained Nicky ‘or being involved in an accident like that…it’s a terrifying moment…it was bad enough going down a pit to earn your living, but to think and worry about, oh, what if that happens again, and I think that’s a hidden disability that has never been taken on.’ (2014). Other participants at the seminar recalled instances where miners who’d witnessed a fatal accident, but were themselves unharmed, subsequently suffered from guilt, taking a long period of absence from work, or never returning. These anecdotes suggest that further oral history work may enable us to tease out the psychological consequences of witnessing accidents, which are often difficult to excavate from other sources. It is noteworthy that participants proffered other people’s stories: a culture of emotional suppression may well inhibit former miners from relaying their own experiences, but perhaps poses less of a barrier when discussing the experiences of others. For many miners, economic circumstances would have necessitated returning to work after experiencing or witnessing an accident, however unpalatable this prospect was: as Ben Curtis and Steven Thompson have recently observed, retirement in the sector typically denoted ‘the inability to continue working as a result of disability’ (2015, p. 591). Alongside the toll of disability and death following accidents, miners would also have been acutely aware of the burden of occupational illnesses, most notable pulmonary diseases, which frequently entailed premature death. As Thompson and Curtis observe, the visible evidence of industrial disability in coalfields community, and the high probability of being afflicted by a progressively disabling occupational illness, would have had ‘profound and multi-layered psychological effects’ (2015, p. 594).

Certainly, discussion around mining accidents and rehabilitation suggest that doctors and officials were aware of a psychological dimension. Rather than educate miners about the possible psychological consequences of experiencing a traumatic event however, the attitude seems to have been to try and conceal this, for fear that miners would exploit it. Doctors TD Spencer and DB Cook, for example, wrote to the chief medical officer for the National Coal Board, the statutory corporation established in 1947 to run the newly-nationalized mining industry, urging that the requirement to specify fractures of the skull when notifying accidents to the inspectorate be removed. Both doctors felt that such fractures gave little indication as to the severity of the head injury, yet news of such an injury had ‘a profound psychological effect’ in the affected miner which hindered recovery (Spencer, 1952). ‘Knowledge of a fractured skill may produce prolonged invalidism in the patient’, explained Cook, ‘especially if encouraged by his union’ (1957). Similarly, Board officials meeting in 1962 expressed concerns regarding laxity in certification amongst colliery medical practitioners, and suggested that it would be worth re-examining the administration of the Board’s sickness and injury schemes to minimize malingering (Wynn et al., 1962). This opens up interesting questions about the relationship between miners, doctors and other officials, and how this might have shaped responses towards, and understandings of, miners’ mental distress.

Another set of sources which might enable us to interrogate the connections between work and mental distress are materials generated by suicide cases. In 1891, the *Shields Daily Gazette* reported on the inquest of a miner named Robert Bell. Robert had worked at Seaham Colliery, where his father had died in an explosion 11 years earlier. He took his life by jumping down a 1800 foot pit shaft (Anonymous, 1891). Judging by other newspaper reports of miners’ suicides, this was a fairly common method (Anonymous, 1892a; Anonymous, 1892b; Anonymous 1893a, 1893b; Anonymous, 1933; Anonymous, 1896^1^). Perhaps on one level this was simply a convenient option. However, the frequency with which miners chose to end their lives on work premises certainly suggests that their work may have had some bearing on their state of mind. Equally, the fatal accident of Robert’s father—like the fatal accident of the father of Patrick Curran, who had been admitted to Woodilee asylum in 1912—is suggestive of a cumulative psychological toll, as miners in close knit communities lost friends and relatives in accidents which they had survived.

Some suicide cases remind us that the psychological impact of accidents could be exacerbated by concerns about future employment prospects or the removal of compensation payments or injury benefits. In 1941, John Charlton, formerly employed by Woodhorn Colliery, committed suicide. John had ceased working a year earlier due to a leg injury caused by an accident at work, and eye strain due to a previous accident. His doctor told an inquest that John’s physical condition had been improving and that his compensation was therefore due to be stopped. According to the doctor, John was ‘worried about how he was going to exist if he came off compensation (Anonymous, 1941). Similar themes emerge in the case of Ellis R, a 57 year old miner who returned home on 14 January 1959 and told his wife Jane that he’d swallowed 200 aspirin, washed down with half a bottle of whisky (Ministry of Pensions, 1959).^2^ Ellis died the following day, and Jane subsequently applied for industrial death benefit, on the grounds that her husband’s death resulted from an accident four months earlier, when he had been hit in the face by a girder while setting a prop. Ellis had been admitted to hospital following the accident and had been in receipt of injury benefit for several months, but had returned to work in December. He was then signed off work after Christmas, first on the grounds of facial injuries, and then due to an influenza type cold.

Jane appealed the local insurance officer’s decision to reject her claim to industrial death benefit, and her daughter told the appeal hearing that her father feared that the accident would prevent him from working again. He began to suffer from insomnia, and sat up at night brooding. He also complained of headaches, and frequently burst into tears. His doctor concluded that he had taken his life ‘in a state of depression and despair for the future, a condition arising from his accident.’ Examining the medical evidence, Dr Aveling, a senior medical officer of the Ministry of Pensions, insinuated that Ellis was a malingering hypochondriac. The injuries Ellis had sustained, he claimed, amounted to little more than a ‘first class black eye’, while Ellis’ sickness record suggested that he was ‘accustomed to fairly long periods of incapacity for not very serious conditions’. Aveling claimed that the diagnosis of Asthenia, which featured in Ellis’ record, ‘simply means weakness but is often used by doctors on certificates for neurasthenia.’ He contended that the influenza type cold Ellis had been signed off with after Christmas had triggered a depression, that many people suffered a first attack of melancholia while in their fifties, and that his suicide had nothing to do with the original accident. Despite Aveling’s evidence, the Commissioner, Sir Robert Micklethwaite, ruled that the suicide was due to the accident.

Ellis’ case illustrates several key themes. First, it demonstrates how an accident could trigger subsequent mental distress, and reveals how the mechanisms of compensation led to the contestation of work-related mental distress. It also reveals that we can locate the mentally distressed miner in sources we might not expect to. For example, Ellis had spent some time at the Miners’ Convalescent Home in Blackpool before his return to work in December 1958, notwithstanding the purported exclusion of cases of mental distress from convalescent homes. Furthermore, Aveling was inclined to interpret the diagnosis of Asthenia as a thinly veiled reference for neurosis. Finally, it is noteworthy that the medical officer evaluating the case made very little effort to conceal his distrust for the narrative put forward by Ellis’ widow. As Joannne Bourke has discussed, anxieties about malingering—consciously simulating symptoms of illness in order to deceive—mushroomed following the Employers’ Liability Act and Workmen’s Compensation Acts, which proffered tangible benefits to those workers who succeeded in their deception, and received further attention during the course of the First World War as military officials sought to identify soldiers who were attempting to shirk combat. In principle, doctors distinguished between malingering and neuroses: the neurasthenic also simulated their symptoms, but did so unconsciously, and therefore lacked the intention to deceive. In practice, there was considerable slippage between the terms, with many commentators arguing that fear underpinned both behaviours, and that it was difficult to distinguish between the two (Bourke, 1996; Bogacz, 1989). Bourke argues that doctors presented both neurasthenia and malingering as a lack of will; forms of infantile regression which were potentially indicative of a flawed hereditary constitution (1996, pp. 76–123). Class-based tensions suffused these debates: middle-class doctors were more inclined to treat patients from a similar background sympathetically, and, conversely, to view working-class patients as malingerers, or as hereditarily predisposed to psychoses or hysteria (Barham, 2004). These ideas linger on today in a modified form, finding expression in the view that occupational stress is a manifestation of personal weakness (Palmer, 2015). In both war and peace time, doctors increasingly argued that one of their functions was to detect and expose malingering (Bourke, 1996).

Because scepticism often pervaded doctors’ accounts when they were presented with occupational illnesses and disabilities which might have a psychological dimension, trade unions felt obliged to focus on the physical components of workers’ illnesses. This becomes apparent if we examine miners’ nystagmus: a common disorder amongst miners, characterized by oscillation of the eyeballs, which by the interwar years had become the leading cause of disability under the Workmen’s Compensation Acts, accounting for more than 65 percent of all cases of industrial disease by 1925 (Home Office, 1926). Initially, doctors had been inclined to attribute this condition to either lighting in mines (Bell Taylor, 1875), or the working position of miners (Snell, 1892). However, in the aftermath of the First World War an alternative hypothesis was advanced: that miners were either faking the condition to secure compensation, (McNamara, 1936) or that the disorder was an occupational neurosis in which miners unconsciously produced symptoms as a means of withdrawing from a terrifying working environment (Culpin, 1933).

In research funded by the British Colliery Owners’ Research Association,^3^ psychiatrist Edward Stern characterized miners’ nystagmus as a psychosomatic disorder which stemmed from miners’ faulty childhood development and flawed personalities (1948, 1950). Miners, Stern asserted, were often ‘dull and backward’, and ‘have always tended to be a peculiar people’, for their occupation ‘involves the spoiling, despoiling, disfiguring, and laying waste of his environment’ (1948, p. 223). He concluded that miners’ nystagmus involved potency fantasies, established in the infant during habit-training, ‘when he can win approval or disapproval by evacuating or withholding his faeces at the proper time’. In Stern’s analogy, miners could win approval or disapproval from the community by ‘delivering or withholding coal from the bowels of the earth. A journey in a mine in the long subterranean, intestine-like galleries, makes this latter analogy particularly appropriate.’ Indeed, Stern insinuated that miners’ trade union activities stemmed from unresolved childhood issues. ‘Most mining disputes’, he noted, ‘arise over the question of production or wages; money being a well-known faecal symbol, familiarly expressed as “filthy lucre”’ (1950, p. 389). Stern’s observations were built on a long-standing pejorative medical narrative which pathologized miners as desensitized brutes with degraded mental faculties who were largely impervious to pain (Turner and Blackie, 2018, pp. 58–59). This perception of miners enabled doctors to interpret industrial disputes as symptomatic of mental disorder: in 1893, Dr Smith, the medical superintendent of Durham County Asylum, suggested in his annual report that a recent dispute in the coal industry had contributed a large proportion of the cases admitted to the asylum in that year. According to the *British Medical Journal*, Dr Smith’s report gave rise to ‘an animated discussion’ at a meeting of the county council, with some opposing the adoption of the report and proposing that an inquiry be launched into the report’s claim that the strike had had an detrimental impact upon the mental condition of the miners (Anonymous, 1893b). Writing in modernist journal *New Age* in 1919, the Scottish educator A.S. Neill similarly argued that class warfare was a symptom of workers’ inferiority complex, requiring a psychological as opposed to an industrial or economic solution (cited in Thomson, 2006, pp. 82–83).

It is perhaps unsurprising that while the TUC’s medical advisor Dr Hyacinth Morgan acknowledged privately that miners’ nystagmus had ‘a neurotic element’, he nevertheless sought to refute such arguments publically, believing this to be the only way to preserve miners’ compensation for this disorder (1936). In a similar vein, while Morgan recognized that accidents at work could cause psychological disturbance, he feared that openly supporting psychiatric services for workers would undermine efforts to secure compensation and support for workers suffering from physical health issues. The difficulty for Morgan lay in creating a terminology which legitimated workers’ mental distress, and successfully differentiated this distress from popular conceptions of mental illness and the language of malingering. In an exchange with one doctor who sought TUC funding to develop psychological rehabilitation services for workers who had suffered accidents, Morgan asked that the words ‘mental rehabilitation’ be substituted with the term ‘injury psychoneurosis (traumatic neurasthenia)’ (Long, 2011, pp. 141–142). Ultimately, this proposal was not taken forward and—in this era at least (Melling, 2014, p. 163)^4^—the TUC believed that the best way to support workers suffering from mental health issues was to dress these problems up as physical illnesses. Such attitudes appear to have exerted a tenacious grip. Nicky Wilson reflected that the National Union of Mineworkers helped secure compensation for workers suffering from psychological distress following accidents in the 1980s, but did so on the grounds of physical health. ‘It was the physical aspect we as a union concentrated on’, Wilson acknowledged, describing mental distress as ‘a hidden thing that years ago was never covered’ (2014).

## Conclusion

In this article, we have used the case study of coal mining to outline how a more expansive history of what we might broadly conceive of as work-related mental distress could be unearthed. While a hunt for primary sources on this topic might initially seem unpromising, scratching just a little further reveals a widespread consensus amongst doctors, employers, workers, trade unions and government bodies that workers did indeed suffer from psychological disorders. These records do not lend themselves to a quantitative reconstruction of the extent of work-related mental distress in the past. Nor do they demonstrate that the current epidemic of occupational stress is simply the rebranding of a long-standing, stable phenomenon. What they do reveal, however, is how these groups debated the cause of these disorders and appropriate responses, and how in turn these debates were shaped by medical, political, economic, occupational and industrial factors.

We also suggest that closer attention to work-related distress in different occupational sectors will allow us to tease out the nuanced role played by the workplace environment, working relationships, and working practices on mental health, to chart how and why understandings and manifestations of work-related mental distress changed, and to examine the relationship between physical and mental health at work. Such an analysis need to examine how these factors intersect with class and gender in both industrial and post-industrial contexts. This article considers a heavy industry; an occupation with a male working-class work force who were viewed by doctors as inclined to malingering; a hazardous working environment and a working culture not attuned to expressions of emotion. This, we argue, played a significant role in constructing a milieu in which miners’ mental health slipped under the radar. How might the story differ if we compare this picture to white collar sectors; to female-dominate professions; to different working environments and working cultures? It does not automatically follow from this that occupations typically identified as female and middle class effectively addressed occupational mental health issues. Nursing, for example, posed a number of health risks to its practitioners, partly because nursing leaders’ desire to establish the occupation as a profession for middle-class women led to an emphasis on vocation which fostered a culture of overwork and downplayed physical and psychological health risks (Palmer, 2014). Women’s perceived greater susceptibility to mental health problems may in practice have obscured the influence of work on female employees’ states of mind.

We have not focused here upon the impact of working relationships on mental health within mining, but this would be a valuable line of inquiry for further research. What impact did varying managerial styles at different pits prior to nationalization have on workers’ emotional resilience, how did nationalization affect this picture, and did the deregulation of working practices which tended to characterize coal mining as the sector contracted undermine mental wellbeing? Employees of the Lothian Coal Company in the first half of the twentieth century, for example, recalled the untrammelled power of the owner, Mungo Mackay, who operated a network of informers and owned most workers’ houses. They described how the threat of eviction generated a serf-like existence and working culture of fear (MacDougall, 1995). We argue here that the physical hazards of coal mining generated a distinctive story of work-related mental distress within this industry, but factors acknowledged to be at work in producing occupational stress in the post-industrial workplace, such as an inability to control the working environment and a mounting workload, could also be significant. In coal mining, these factors are likely to have risen to the fore as the sector went into decline, and could also militate against physical health and safety. Indeed, this serves to reinforce our point that the physical and emotional hazards of mining were interconnected.

What impact do changing working practices and economic circumstances have on mental health – not to mention the decline of certain occupational sectors, such as mining, and the rise in unemployment? This article has focused primarily on the hazards posed by work to mental health, but has also examined cases in which concerns about future employment prospects produced anxiety and distress. Work has been deployed in varying ways as a therapeutic and protective agent in mental healthcare for over two hundred years (Ernst, 2016), while studies analysing the detrimental impact of unemployment on mental wellbeing dates back to at least the interwar depression (Beales and Lambert, 1934; Jahoda, Lazarsfeld and Zeisel, 1972). Surveying embodied experiences of deindustrialization, Arthur McIvor highlights how loss of employment and precarious work had a particularly pernicious impact upon many working-class men, whose identities were intimately bound up in their performance of heavy industrial work, and their breadwinner status. Faced with this challenge to their identity, some abused drugs or alcohol, and mental health problems proliferated. Following layoffs at Barony Pit in Ayrshire in 1962, three miners killed themselves (McIvor, 2017, pp. 28–31). As men are typically more reluctant than women to seek treatment for mental health issues, and mental health services are currently skewed towards detecting women’s problems (Haggett, 2015) there is a considerable risk that the psychological impact of deindustrialization on men will only be partially addressed, entrenching long-term morbidity in post-industrial regions.

Alongside the risks posed by monotonous industrial work detailed by industrial psychologists, and the recent rise of stress-related illnesses in service sectors, we believe that the hazardous environments faced by many workers in heavy industries such as mining generated their own psychological consequences, which were overlooked. While such hazards might seem abstract in Britain’s current labour market, they remain pertinent in a globalizing world where many workers still engage in hazardous work.

## Footnotes

1 In this instance, the deceased jumped into the colliery pond which supplied the engine boilers with water.

2 Debate hinged on whether or not Ellis’s suicide was voluntary or involuntary—i.e., whether the pain resulting from the accident drove him out of his mind to the extent that he felt compelled to take his life.

3 Established in 1924, the work of the Association was jointly funded by the coal industry and the Department of Scientific and Industrial Research.

4 Melling discusses how the TUC began to examine the issue in the 1970s.
